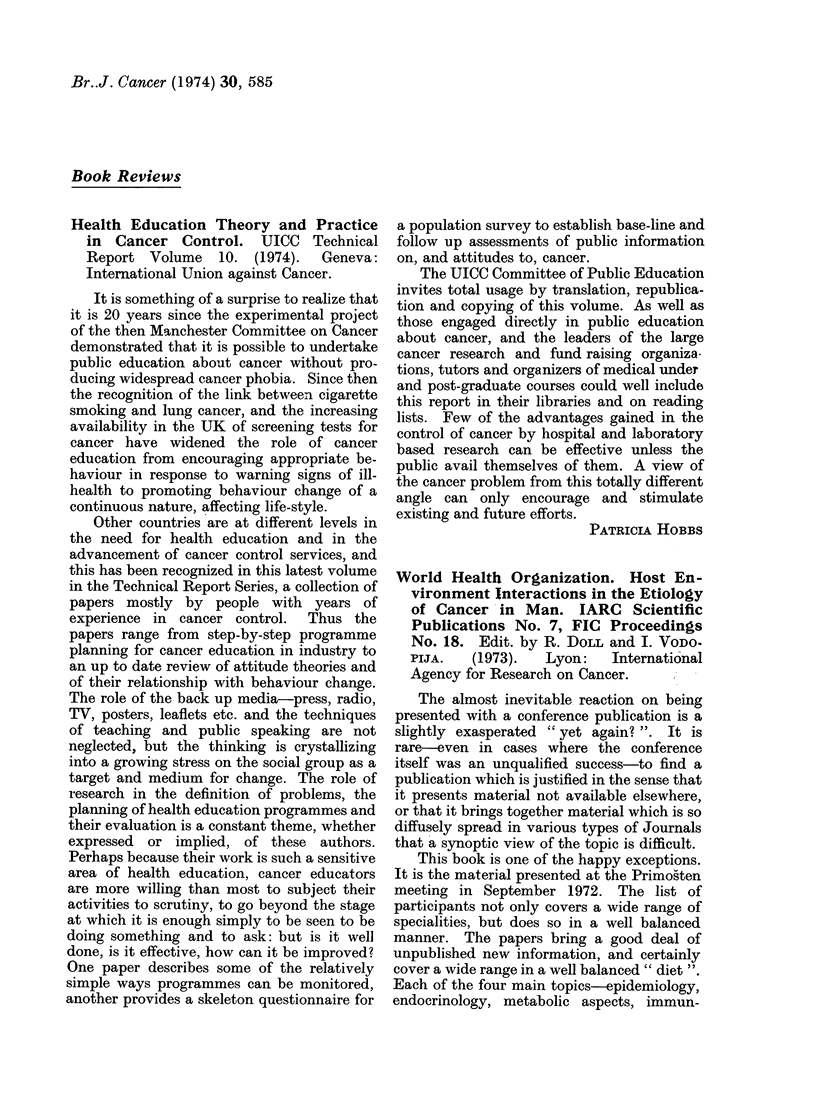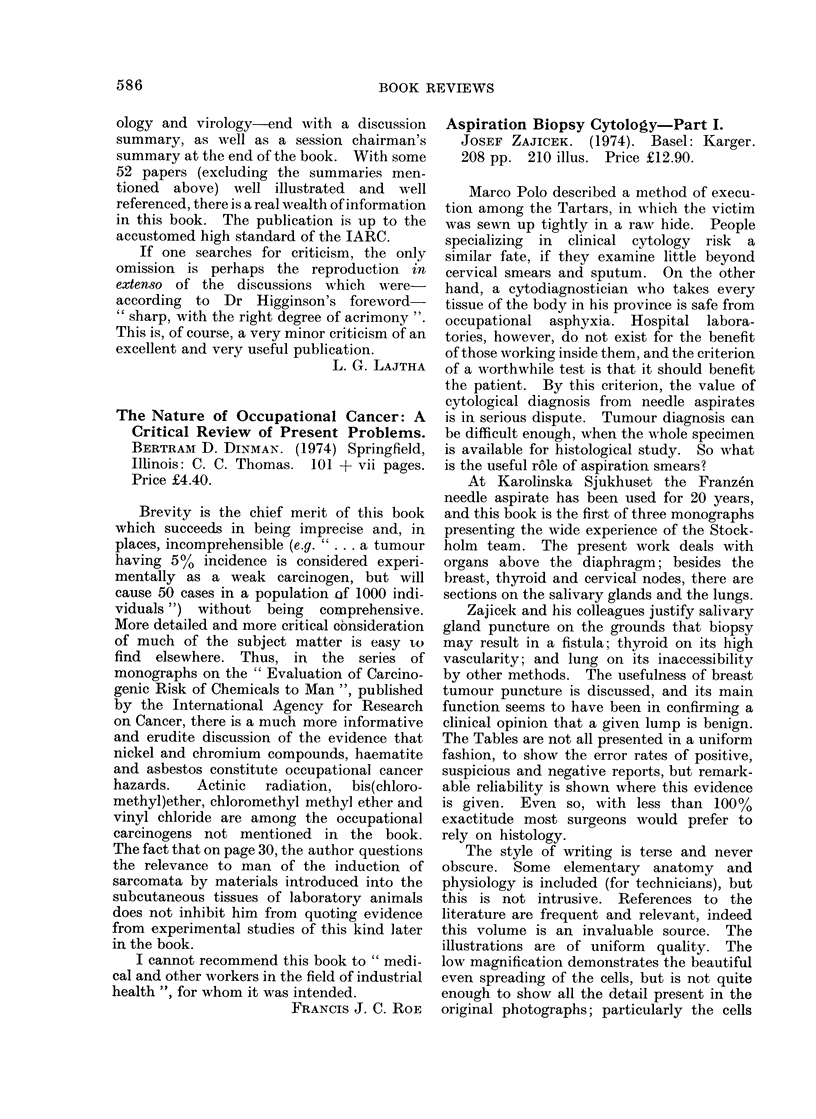# World Health Organization. Host Environment Interactions in the Etiology of Cancer in Man. IARC Scientific Publications No. 7, FIC Proceedings No. 18

**Published:** 1974-12

**Authors:** L. G. Lajtha


**World Health Organization. Host Environment Interactions in the Etiology of Cancer in Man. IARC Scientific Publications No. 7, FIC Proceedings No. 18.** Edit. by R. DOLL and I. VODOPIJA. (1973). Lyon: International Agency for Research on Cancer.

The almost inevitable reaction on being presented with a conference publication is a slightly exasperated "yet again?". It is rare—even in cases where the conference itself was an unqualified success—to find a publication which is justified in the sense that it presents material not available elsewhere, or that it brings together material which is so diffusely spread in various types of Journals that a synoptic view of the topic is difficult.

This book is one of the happy exceptions. It is the material presented at the Primošten meeting in September 1972. The list of participants not only covers a wide range of specialities, but does so in a well balanced manner. The papers bring a good deal of unpublished new information, and certainly cover a wide range in a well balanced "diet". Each of the four main topics—epidemiology, endocrinology, metabolic aspects, immunology and virology—end with a discussion summary, as well as a session chairman's summary at the end of the book. With some 52 papers (excluding the summaries mentioned above) well illustrated and well referenced, there is a real wealth of information in this book. The publication is up to the accustomed high standard of the IARC.

If one searches for criticism, the only omission is perhaps the reproduction *in extenso* of the discussions which were—according to Dr Higginson's foreword—" sharp, with the right degree of acrimony ". This is, of course, a very minor criticism of an excellent and very useful publication.

L. G. LAJTHA